# The impact of disasters on maternal mental health: a systematic review

**DOI:** 10.7189/jogh.16.04126

**Published:** 2026-04-17

**Authors:** Minnat Seema, Clifford Afoakwah, Elcin Tuzel, Joshua Byrnes

**Affiliations:** 1 Centre for Applied Health Economics, Griffith University, Nathan, Australia; 2 Australian Centre for Health Services Innovation, School of Public Health and Social Work, Queensland University of Technology, Kelvin Grove, Australia; 3 Metro North Health, Jamieson Trauma Institute, Herston, Australia; 4 Centre for the Business and Economics of Health, The University of Queensland, St Lucia, Australia

## Abstract

**Background:**

The vulnerability of maternal mental health is increasing with the frequent increase in natural and man-made disasters, yet evidence remains fragmented. We aimed to synthesise quantitative evidence on the impact of disaster exposure on maternal mental health and related pregnancy outcomes.

**Methods:**

We systematically searched 12 electronic databases, nine sub-databases, and additional web sources for the studies published between 1995 and 2025. We included studies that assessed the impact of disaster exposure during pregnancy and provided quantitative outcomes for depression, anxiety, stress, posttraumatic stress disorder (PTSD), or adverse pregnancy outcomes.

**Results:**

Of 18 604 studies, 24 studies met the inclusion criteria. Natural disasters were the highest group (n = 16), including earthquakes (n = 8), floods/cyclone/hurricane (n = 7), and wildfire (n = 1), followed by man-made disaster (n = 7), including conflict/war/terrorism (n = 5), nuclear disaster (n = 1), plane crash (n = 1), and war (n = 1), and one hybrid disaster. Disaster showed a consistent increase in the risk of depression, anxiety, stress, and PTSD among pregnant women, with effects persisting up to six years post-disaster event. Across studies, disaster exposure increased maternal mental health problems by approximately 30% to over 200%, and PTSD odds increased seven to 10-fold among highly exposed pregnant women. Three studies reported that both depression and PTSD during pregnancy were associated with adverse pregnancy or early child outcomes, suggesting potential intergenerational effects.

**Conclusions:**

Disasters exert enduring and profound impacts on maternal mental health, with consequences cascading across both mother and child. Urgent, cost-effective and disaster-informed policies are needed to mitigate such long-term impacts.

**Registration:**

PROSPERO: CRD42023445559.

Mental health constitutes a vital dimension of overall well-being that enables individuals to manage life’s challenges, realise their potential, and actively contribute to society. It is more than the absence of mental disorders; mental health exists on a continuum and is shaped by various psychological, biological, and environmental factors [1].

Understanding mental health as a dynamic and multifactorial construct is essential to addressing the unique vulnerabilities of specific populations, particularly during periods of heightened stress such as pregnancy and environmental disasters. Women face twice the risk of mental illness compared to men [2], and pregnant women are among the most vulnerable. Approximately one in five women experiences a mental health condition during pregnancy or within the first year postpartum [3]. The estimated prevalence of anxiety during pregnancy varies, with meta-analyses suggesting rates between 8.5% and 15.2% during pregnancy and around 9.9% postpartum [4].

These challenges intensify during environmental crises such as disasters, where pregnant women face heightened risks that may also affect the health of their unborn children [5]. Additionally, the lack of secure and safe environments during disasters exacerbates stress and anxiety, as some studies report a positive relationship between anxiety, stress, and depression among pregnant and postpartum women living in earthquake-affected areas [6]. Perinatal stress has been linked to adverse outcomes such as preterm birth and low birth weight (LBW), which can have long-term effects on child’s health and development [7]. Pregnant women in disaster-affected areas often face a difficult balance between coping with the disaster and managing their pregnancy, often feeling overwhelmed and unable to fully focus on their pregnancy because of the crisis, while at the same time being forced to confront its implications for their child.

A growing body of research has examined trajectories of distress following traumatic events. The identified trajectories include resilient, recovery, and chronic patterns across a diverse range of outcomes, such as posttraumatic stress disorder (PTSD) and depression [5,7–10]. Previous studies have explored the maternal mental health effects resulting from exposure to natural disasters [11], or man-made disasters [12] or specific events like earthquakes, hurricanes, and war [7,13–16]. However, no study has systematically synthesised the impacts of exposure to natural, man-made or hybrid disasters on maternal mental health. Therefore, we aimed to synthesise quantitative evidence of the mental health of the women who were in any trimester of pregnancy at the time of disaster occurrence. Such knowledge is critical for the comparative evaluation of the heterogeneous impacts of the different forms of disasters on pregnant women while enhancing the development of support systems for pregnant women during disasters.

## METHODS

### Protocol and registration

We conducted a systematic review in accordance with the PRISMA 2020 statement (Table S6 in the **Online Supplementary Document**) [17], and registered it with the International Prospective Register of Systematic Reviews (PROSPERO: CRD42023445559).

### Eligibility criteria

We included studies that examined the impact of natural (*e.g.* earthquakes, floods, hurricanes, and wildfires) or man-made (*e.g.* war, terrorism, armed conflict, and industrial accidents) disasters, defined according to Centre for Research on the Epidemiology of Disasters categories [18], on the mental health of pregnant women. We included quantitative experimental, quasi-experimental, cohort, case-control, and cross-sectional studies that reported measurable mental health outcomes during or after disaster exposure.

We excluded studies that did not measure disaster exposure, did not report mental health outcomes relevant to the review, or did not allow pregnant women to be analysed separately. We also excluded qualitative studies, books, reviews, commentaries, editorials, conference abstracts, case reports, grey literature, and other non-peer-reviewed material since they did not provide quantitative effect estimates suitable for synthesis. The search covered 1995–2025, reflecting the period in which validated maternal mental health instruments (*e.g.* Edinburgh Postnatal Depression Scale (EPDS), Depression Anxiety Stress Scales, Kessler Psychological Distress Scale, Posttraumatic Stress Disorder Checklist (PCL), and State-Trait Anxiety Inventory) and standardised disaster-classification frameworks became widely adopted, rendering earlier studies methodologically incompatible with our criteria [19]. We only included English-language publications because translation resources were not available and the mental health measurement tools used across studies require validated language-specific versions.

### Information source

We developed the search strategy with input from a professional librarian [20] and followed established systematic review guidelines [21]. We did not consult external subject-matter experts during the development of the search terms. We searched MEDLINE, PsycInfo, CINAHL, Embase, Scopus, Web of Science, ProQuest Central, EconLit, JSTOR, PTSDpubs, Sociological Abstracts, International Bibliography of the Social Sciences, and Google Scholar (Table S1 in the **Online Supplementary Document**).

### Search strategy

We developed a structured Boolean search combining Medical Subject Headings terms and keywords related to pregnancy, mental health outcomes, and disaster types in MEDLINE and adapted it for other databases (Table S2 in the **Online Supplementary Document**). The search execution period of 1 October 2023 to 31 May 2025 reflects the timeframe during which we conducted and updated the review, with automated alerts activated during this period to capture newly published studies.

### Data management and study selection

We imported all search results into Covidence (Veritas Health Innovation, Melbourne, Australia) for automated de-duplication and initial organisation. We screened titles and abstracts against the predefined exclusion criteria and sorted non-eligible records accordingly. We exported the remaining studies to a consolidated EndNote library, where we created subgroups to reflect specific exclusion reasons. When studies met multiple exclusion criteria, we classified them in the following order of priority: subject area, originality, and clarity.

MS and CA independently screened the studies, with JB overseeing the process and resolving any disagreements. MS, CA, and JB jointly verified data extraction for accuracy and completeness. Screening proceeded in two stages: first, we assessed titles and abstracts of original research on disaster-related maternal mental health, and second, we retrieved and evaluated full texts of potentially relevant studies to confirm disaster exposure and eligible mental health outcomes (Table S3 in the **Online Supplementary Document**).

### Data synthesis

We used the population, intervention, comparison, and outcome framework to synthesise the extracted data. ‘Population’ was women who were pregnant at the time of disaster exposure and ‘intervention/exposure’ was any natural or man-made disaster classified using the Centre for Research on the Epidemiology of Disasters system. and Comparison group consisted of pregnant women who were not exposed to disaster or with lower levels of exposure with mental health being the main outcome measure (Table S4 in the **Online Supplementary Document**). Mental health outcomes included depression, anxiety, PTSD, stress, or psychological distress assessed using validated quantitative tools such as the EPDS, Depression Anxiety Stress Scales, Kessler Psychological Distress Scale, PCL and State-Trait Anxiety Inventory, using the Joanna Briggs Institute Critical Appraisal Checklists for quantitative study designs (Table S5 in the **Online Supplementary Document**) [22]. We selected these outcomes because they represent the most consistently assessed and clinically relevant indicators of maternal mental health in disaster research, and the use of validated instruments ensured psychometric reliability and comparability across studies. We only extracted validated quantitative outcomes where reported, however, we only included studies with extractable effect estimates and corresponding confidence intervals (CIs) (n = 13) in the forest plot displaying effects on a logarithmic scale to aid comparability. The forest plot visually summarises the direction and relative magnitude of disaster-related mental health effects. All 24 included studies contributed to the overall synthesis, while we incorporated those lacking extractable effect estimates through conceptual/narrative synthesis. We narratively summarised mental health outcomes measured using non-validated or ad hoc tools but excluded them from the quantitative harmonisation.

Since studies in the synthesis used diverse mental-health tools and reporting formats, we did not statistically standardise individual scale scores, instead, we used a conceptual harmonisation approach by capturing all available quantitative indicators, including prevalence, mean scores, adjusted odds ratios (aORs), relative risks, standardised mean differences, and CIs, and prioritising these summary measures to enhance comparability across heterogeneous study designs and outcome scales. This harmonisation referred to comparing the direction and magnitude of associations across studies rather than standardising raw scores, allowing outcomes from different validated tools to be interpreted within a common conceptual framework. It was piloted on a small subset of eligible studies to ensure clarity and completeness. We made minor refinements during this process to improve variable definitions and consistency before full data extraction commenced.

Therefore, we performed a structured narrative synthesis following guidance from Popay and colleagues [23] and the Cochrane Consumers and Communication Review Group [24]. We managed heterogeneity through structured subgrouping, where we synthesised studies within disaster-type categories and compared them on the direction and magnitude of their reported associations. We first grouped the studies by broader disaster category (natural or man-made) and then by specific type (*e.g.* earthquake, flood/hurricane, conflict, and industrial accident). Within each group, we summarised the findings according to the direction and magnitude of associations using standardised indicators such as aORs, relative risks, regression coefficients, or prevalence estimates. We considered precision of estimates and contextual characteristics (*e.g.* severity of exposure, displacement, socioeconomic factors, timing of measurement) when interpreting variability across studies. We extracted the missing or incomplete reporting as provided, noted explicitly, and interpreted cautiously, and did not exclude studies on this basis. We did not conduct a meta-analysis due to substantial heterogeneity in study design (cohort, cross-sectional, case-control), disaster definitions, mental health measures, and timing of assessment. We did not engage external experts for formal expert review. Instead, senior members of the research team (CA and JB) internally provided expert oversight. Their expertise in quantitative evidence synthesis and disaster-related maternal health informed the validation of eligibility decisions, data extraction, subgrouping approach, and interpretation of heterogeneous findings throughout the narrative synthesis.

## RESULTS

### Summary of systematic review

**Figure 1:**
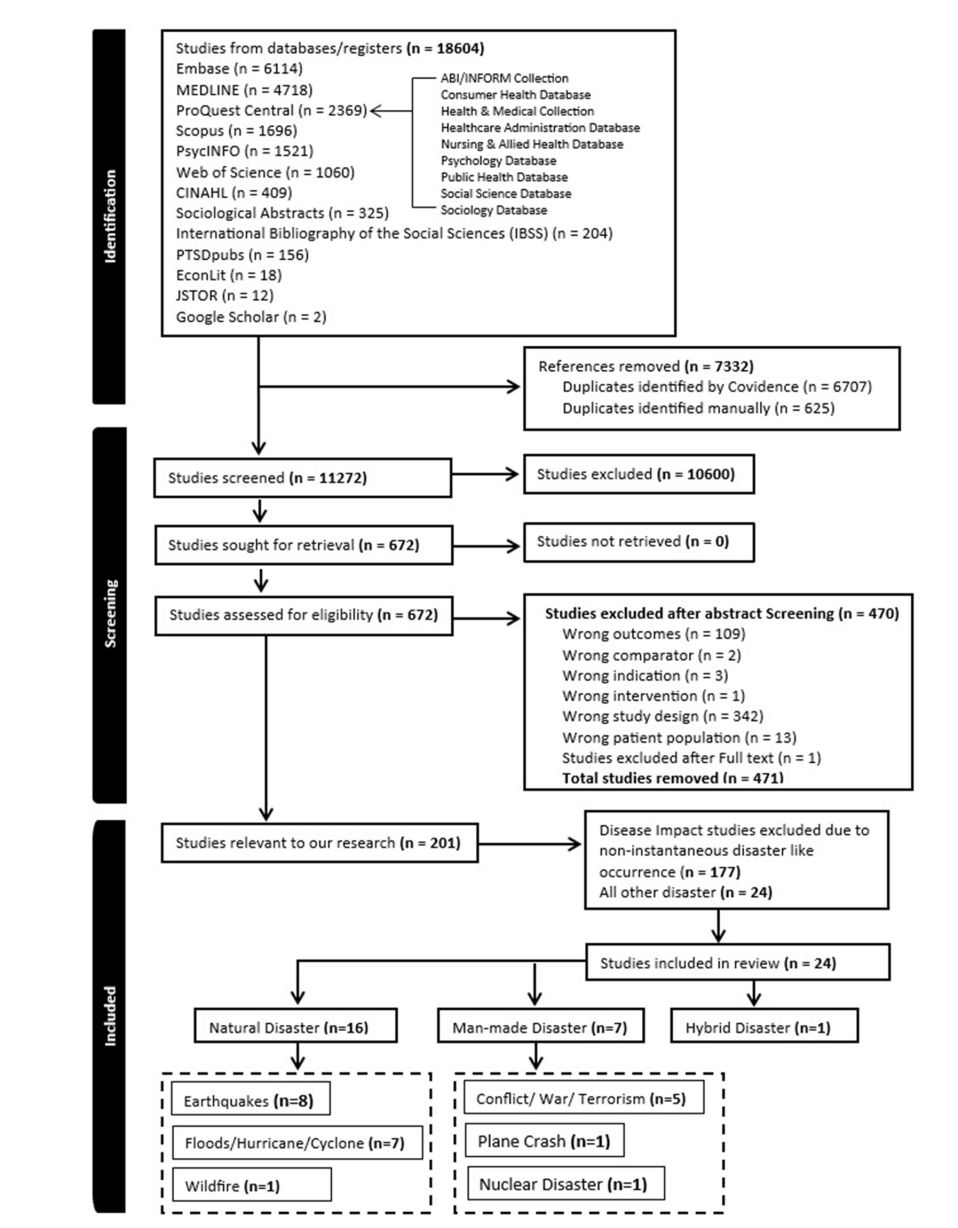
Flowchart of the systematic review.

We identified 18 604 articles across 22 databases and sub-databases. After removing 6707 duplicates in Covidence and 625 manually, we excluded 7332 articles (**Figure 1**). We excluded an additional 10 600 records through title screening. Of the remaining 672 abstracts assessed, we excluded 471, of which we removed 177 pandemic-related studies (*e.g.* COVID-19, 1957 influenza) as they did not meet the disaster criteria. Lastly, we excluded one additional article trough full-text review, resulting in 24 included studies (**Tables 1–3**).

**Table 1:** Summary of the included studies on natural disasters

Study ID	Disaster category	Population	Exposure (disaster) and design	Comparison	Outcomes and key estimates (95% CI)*	Key interpretation
Xiong *et al.*, 2010 [7]	Floods/hurricane/cyclone related maternal mental health outcomes	Pregnant women in New Orleans and Baton Rouge	Hurricane Katrina – prospective cohort	Women with low/minimal exposure	PTSD: aOR = 16.8 (2.6–106.6); depression: aOR = 3.3 (1.6–7.1)	Extremely elevated risk of PTSD when exposure was high
Oni *et al*., 2015 [14]	Floods/hurricane/cyclone related maternal mental health outcomes	Pregnant women exposed to Katrina stressors	Hurricane Katrina – cross/longitudinal	Variation in perceived stress	Perceived stress‏ – induction: aOR = 1.50 (1.34–1.99); GDM: aOR = 1.13 (1.02–1.25)	Psychological distress links to adverse maternal outcomes (and by implication mental health)
Paquin *et al*., 2021 [25]	Floods/hurricane/cyclone related maternal mental health outcomes	Pregnant women in Queensland floods	Queensland Floods – longitudinal	Internal comparison by distress/appraisal level	Peritraumatic distress × negative appraisal – anxiety: β = 0.52 (SE = 0.15; *P* < 0.001); depression: β = 0.55 (SE = 0.17; *P* < 0.01)	Subjective reaction (distress + appraisal) mattered more than objective hardship
Brock *et al*., 2015 [26]	Floods/hurricane/cyclone related maternal mental health outcomes	Pregnant women exposed to Iowa floods	Iowa Floods – prospective cohort	Lower flood exposure	Indirect effect (mediation) of flood severity via distress – depression: β ≈ 0.16 (0.04–0.30)	Mediating role of peritraumatic distress in the disaster-mental-health link
Parayiwa *et al*., 2023 [15]	Floods/hurricane/cyclone related maternal mental health outcomes	Pregnant women exposed to Queensland cyclones	Queensland Cyclones – cross-sectional	Non/less-exposed pregnant women	Perceived stress: β = 0.48 (*P* = 0.014) for New Zealand-born + cyclone stressors	Disaster-related stress remains elevated in pregnant women and influenced by demographic background
Harville *et al*., 2023 [27]	Floods/hurricane/cyclone related maternal mental health outcomes	Pregnant women before/after Hurricane Michael	Hurricane Michael – pre/post design	Pregnant before vs after storm	Elevated depression screening: OR ≈ 1.4 (1.1–1.8; *P* < 0.01)	Post-disaster pregnancy period showed increased screening scores for mental health issues
Simeone *et al*., 2021 [10]	Floods/hurricane/cyclone related maternal mental health outcomes	Pregnant during Irma/Maria or during recovery	Hurricanes Irma and Maria – population-based cross-sectional	Pregnant at landfall vs pregnant in recovery period	Postpartum depressive symptoms: feeling unsafe aPR = 2.4 (1.2–4.9); difficulty obtaining food aPR = 2.1 (1.1–4.1); other stressors aPR ≈ 1.7–2.1	Material hardship and perceived insecurity during and after the hurricanes substantially increased postpartum depression risk
Qu *et al*., 2012 [28]	Earthquake-related maternal mental health outcomes	Pregnant women in Sichuan	2008 Sichuan (China) Earthquake – cross-sectional	Varying levels of exposure (bereavement, witnessing horror)	Bereavement/horror – PTSD OR = 1.80 (1.43–2.26); pregnancy-stress – depression/PTSD OR = 1.19 (1.12–1.27); family support protective OR = 0.84 (0.73–0.98)	Earthquake-related trauma and pregnancy-specific stress substantially increased PTSD and depression risk
Dong *et al*., 2013 [29]	Earthquake-related maternal mental health outcomes	Pregnant women in Sichuan	2008 Sichuan (China) Earthquake – comparative cross-sectional	Earthquake-affected vs non-affected county	Antenatal depression: 34.5% vs 39.6% (CIs overlapping); medium vs high husband support – higher depression (*P* < 0.05)	No residual area effect, but psychosocial stressors strongly predicted depression
Ren *et al*., 2014 [30]	Earthquake-related maternal mental health outcomes	Pregnant women at the epicentre	2013 Lushan (Japan) Earthquake – cross-sectional	Differences in coping style and support	Depression 35.2%; Injured relatives, low support, negative coping significant predictors (all *P* < 0.05)	Direct exposure plus poor coping/support markedly increased depressive symptoms
Hibino *et al*., 2009 [31]	Earthquake-related maternal mental health outcomes	Pregnant/postpartum women in Noto Peninsula	Noto Peninsula (Japan) Earthquake – panel study	Internal comparison across resilience levels (SOC)	EPDS≥9: 13.1%; Anxiety about earthquakes – higher EPDS (β = 0.27; *P* = 0.01); SOC moderated effect (β = −0.21; *P* = 0.02)	Earthquake anxiety significantly worsened symptoms, buffered by strong stress-resilience traits
Tanoue *et al*., 2021 [16]	Earthquake-related maternal mental health outcomes	JECS cohort: pregnant women in Miyagi	2011 GEJE – prospective cohort	Miyagi vs 13 less-affected regions	Psychological distress: aORs = 1.38–1.92 across 3 y (all 95% CIs exclude 1)	Large cohort evidence shows sustained multi-year distress elevation after a major earthquake
Khatri *et al*., 2019 [32]	Earthquake-related maternal mental health outcomes	Pregnant women in Nepal	2015 Nepal Earthquakes – cross-sectional	Comparison by structural damage, IPV, and support	CMD symptoms (>12): 21.9% (95% CI = 18.4–25.8); earthquake damage and IPV increase symptoms; partner support protective (all *P* < 0.05)	Structural damage and violence amplified CMD risk, while supportive relationships buffered it
Watanabe *et al*., 2016 [33]	Earthquake-related maternal mental health outcomes	JECS cohort: pregnant women in Miyagi	Miyagi (Japan) after GEJE – population cohort	Disaster vs non-disaster regions	Persistently higher psychological distress ORs = 1.67–2.19 (CIs exclude 1)	Earthquake effects persisted even in inland areas without tsunami damage
Yalniz *et al*., 2024 [6]	Earthquake-related maternal mental health outcomes	Pregnant/postpartum women in Türkiye	Türkiye 2023 Earthquakes – cross-sectional	Tent-city residents vs community dwelling	Stress, anxiety, depression highly correlated (all *P* < 0.001)	Early post-disaster period shows high psychosocial burden among displaced pregnant women
Verstraeten *et al*., 2020 [34]	Wildfire-related maternal mental health outcomes	Pregnant and peri-conception women in Fort McMurray	Fort McMurray 2016 Wildfire (Canada) – longitudinal cohort	Internal comparison by levels of peritraumatic distress and social support	Possible PTSD: 23.5%; probable PTSD: 26%; peritraumatic distress – PTSD symptoms: r = 0.56, *P* < 0.001; social support protective: β = 7.08 (95% CI = 2.29–11.87; *P* = 0.004); distress × support interaction: β = 0.217 (95% CI = 0.049–0.386; *P* = 0.012)	Wildfire-related peritraumatic distress strongly predicts PTSD symptoms, with social support offering only partial buffering

aOR – adjusted odds ratio, aPR – adjusted prevalence ratio, CI – confidence interval, CMD – common mental disorders, EPDS – Edinburgh Postnatal Depression Scale, GDM – gestational diabetes mellitus, GEJE – Great East Japan Earthquake, IPV – intimate partner violence, JECS – Japan Environment and Children's Study, K6 – Kessler Psychological Distress Scale, OR – odds ratio, PTSD – posttraumatic stress disorder, SE – standard error, SOC – sense of coherence

*Effect estimates correspond to adjusted models where available; prevalence values represent proportions scoring above validated cut-offs.

**Table 2:** Summary of the included studies on man-made disasters

Study ID	Disaster category	Population	Exposure (disaster) and design	Comparison	Outcomes and key estimates (95% CI)	Key interpretation
Seidi *et al*., 2022 [35]	Conflict/war/terrorism related maternal mental health outcomes	Pregnant and ≤1-y postpartum Yazidi internally displaced women in Iraq	ISIS genocide and displacement (Iraq) – Cross-sectional	Internal comparison by sociodemographic and pregnancy-related factors	Health problems: depression risk OR = 3.22 (1.08–9.61); unmarried: OR = 16.00 (1.14–224.28); age protective: OR = 0.84 (0.75–0.94)	Exceptionally high depression burden (78% EPDS>10) among genocide-survivor pregnant women, with strong risk gradients
Muhammad Naseem *et al*., 2015 [13]	Conflict/war/terrorism related maternal mental health outcomes	Pregnant women exposed to armed conflict in Swat Valley (Pakistan)	Armed conflict in Swat Valley (Pakistan) – Cross-sectional	Varying number of PTEs	≥3 conflict-related PTEs: psychological distress OR = 2.62 (1.22–5.61); low perceived family support protective OR = 0.91 (0.88–0.95)	Psychological distress rises sharply once ≥3 traumatic events are experienced
Rashid *et al*., 2020 [36]	Conflict/war/terrorism related maternal mental health outcomes	Mothers of LBW vs normal-weight infants (Pakistan)	Armed conflict and displacement Malakand Division (Pakistan) – case–control	LBW vs normal-birthweight groups	Maternal PTSD strongly associated with LBW: OR = 7.52 (4.02–14.07)	Conflict-related maternal PTSD is a major obstetric risk factor, linked to markedly higher odds of LBW
Silove *et al*., 2015 [37]	Conflict/war/terrorism related maternal mental health outcomes	Pregnant and postpartum women (3–6 mo) in Timor-Leste	Mass conflict and human rights trauma – cross-sectional	Internal comparison by trauma severity	PTSD – depression β = 0.40 (*P* < 0.001); Continuing adversity – depression β = 0.22 (*P* < 0.05)	PTSD and ongoing conflict-related adversity strongly drive antenatal and postpartum depressive symptoms
Makhseed *et al*., 1999 [38]	Conflict/war/terrorism related maternal mental health outcomes	Pregnant women receiving care pre- vs post-Gulf War (Kuwait)	Gulf War invasion (Kuwait) – retrospective time-series	Pre-war vs post-war pregnancies	Increased PIH, pre-eclampsia, miscarriage; mental health not directly measured	Suggests broad perinatal vulnerability during wartime, though mental-health effects are inferred not quantified
Truijens *et al*., 2017 [39]	Plane crash-related maternal mental health outcomes	Pregnant women at 32 weeks’ gestation	MH17 plane crash (Netherlands) – natural experiment (longitudinal cohort)	Season-matched pregnant women at 32 weeks in 2013 (unexposed cohort)	Depressive symptoms (EDS): x̄ = 5.21 vs 4.11; *t =* 2.12, *P* = 0.03; Cohen’s *d* ≈ 0.3; Adjusted association with MH17 exposure *P* = 0.02 (95% CIs not reported)	Plane-crash exposure was associated with a significant short-term rise in third-trimester depressive symptoms
Goto *et al*., 2015 [40]	Nuclear disaster-related maternal mental health outcomes	Pregnant and postpartum women in Fukushima Prefecture	Fukushima nuclear accident (Japan) – population-based cohort	Non-evacuated, low-concern pregnant/postpartum women	Depressive symptoms: evacuation aOR = 1.60 (1.30–1.99); aOR = 1.44 (1.14–1.82); radiation worry aOR = 3.41 (2.59–4.50); 2.32 (1.44–3.76)	Evacuation and radiation-related concern significantly increased maternal depressive symptoms

aOR – adjusted odds ratio, CI – confidence interval, EDS – Edinburgh Depression Scale, EPDS – Edinburgh Postnatal Depression Scale, LBW – low birth weight, MH17 – Malaysia Airlines Flight 17, OR – odds ratio, PIH – post-inflammatory hyperpigmentation, PTE – potentially traumatic event, PTSD – posttraumatic stress disorder, SEM – structural equation modelling, x̄ – mean

**Table 3:** Summary of included study assessing nuclear disaster-related maternal mental health outcomes

Study ID	Population	Exposure (disaster) and design	Comparison	Outcomes and key estimates (95% CI)	Key interpretation
Levey *et al*., 2018 [12]	Pregnant women in Peru	Hybrid disaster context – cumulative trauma including disasters, political violence, assault (Peru) – cross-sectional cohort	Women with fewer or no traumatic events	PTSD (PCL-C≥26): each additional trauma type* aOR = 1.46 (1.39–1.54); 4–6 trauma types aOR = 2.58 (2.16–3.07); ≥7 trauma types aOR = 9.33 (5.82–14.97); adult sexual assault aOR = 4.06 (3.03–5.43); childhood abuse aOR = 3.43 (2.89–4.07)	Cumulative hybrid-disaster trauma produces a steep, dose-response increase in PTSD risk during pregnancy

aOR – adjusted odds ratio, CI – confidence interval, PCL-C – PTSD Checklist–Civilian Version, PTSD – posttraumatic stress disorder

*Trauma categories include interpersonal, disaster-related, and political violence exposures.

**Figure 2:**
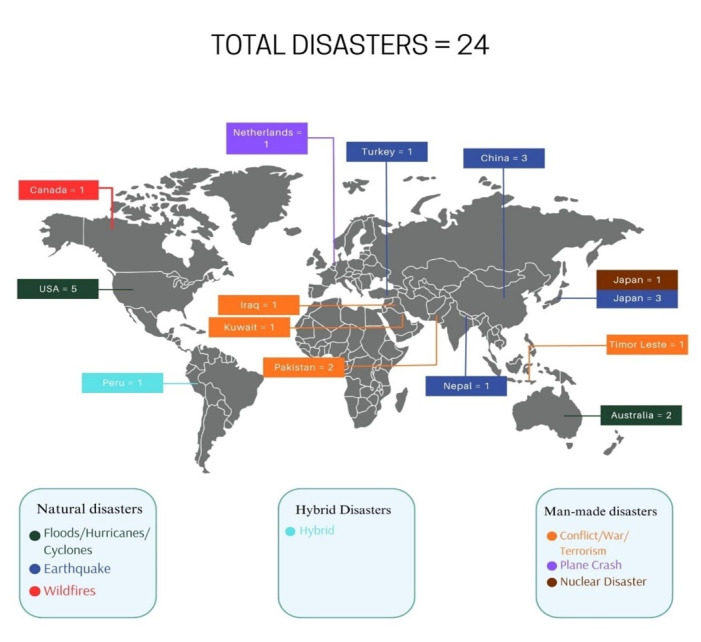
Locations of the included articles reporting disaster occurrence.

Of the included articles, 16 (67%) reported natural disasters, seven (29%) reported man-made disasters, and one (4%) reported hybrid disaster. More than half (n = 14; 58%) of the included articles were from high-income countries, six (25%) from upper-middle-income countries, and four (17%) from lower-middle-income countries (**Figures 2** and Figure S1 in the **Online Supplementary Document**).

**Figure 3:**
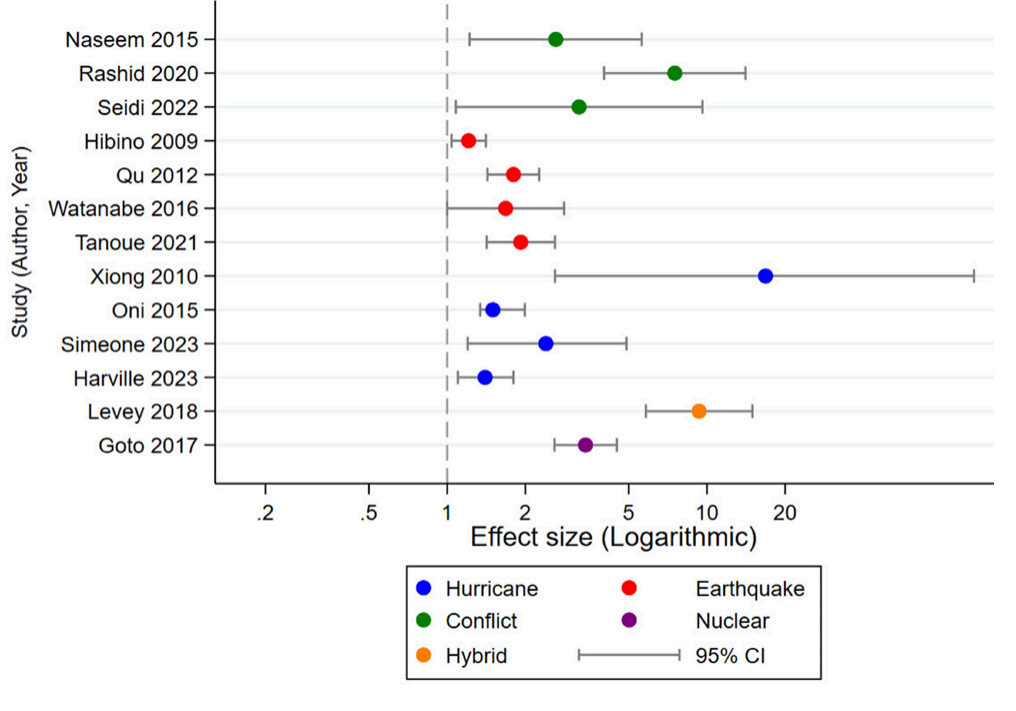
Estimated effect sizes of disaster exposure and maternal mental health. CI – confidence interval.

Out of the 24 included studies, only 13 reported complete quantitative effect estimates with corresponding CIs. We used these studies to construct an exploratory forest plot to visualise the pattern and magnitude of disaster-related mental health effects (**Figure 3**). We did not include the remaining studies in the forest plot due to missing or non-comparable effect-size reporting or insufficient quantitative details. Variability in study design and outcome reporting, along with missing effect estimates in several studies, further limited quantitative synthesis and required cautious interpretation of findings.

Across the extractable studies, disaster exposure consistently increased the risk of adverse maternal mental health outcomes, with most effect sizes lying above the null and several reaching two- to 10-fold elevations. Conflict-related exposures produced the largest effects, followed by hurricanes, earthquakes, and nuclear events. Despite differences in context and measurement, the overall pattern showed a clear and directionally consistent signal: disaster exposure substantially heightened depression, anxiety, stress, and PTSD risk during pregnancy.

### Natural disaster and maternal mental health

#### Floods/hurricane/cyclone and maternal mental health

Results from seven studies showed that pregnant women exposed to floods, hurricanes, or cyclones face significantly elevated risks of adverse mental health outcomes, particularly PTSD, depression, anxiety, and elevated perceived stress (**Table 1**). These studies focused on Hurricane Katrine in the USA (2005) (n = 2), Hurricane Irma and Maria in the USA (2017) (n = 1), the Queensland floods in Australia (2010–2011) (n = 2), the Iowa Flood in the USA (2008) (n = 1), and Hurricane Michael in the USA (2018) (n = 1). In a landmark prospective cohort of women pregnant during Hurricane Katrina recruited at antenatal clinics in New Orleans and Baton Rouge, Xiong and colleagues [7] found that women with ‘high hurricane exposure’ (≥3) had significantly higher odds of PTSD (aOR = 16.8; 95% CI = 2.6–106.6, *P* < 0.05) and depression (aOR = 3.3; 95% CI = 1.6–7.1, *P* < 0.05) compared to women without high exposure.

In a related study [14] of 146 pregnant women exposed to Hurricane Katrina, the researchers assessed coping styles with the Carver’s Brief Coping Orientation to Problems Experienced Inventory, a 28-item self-report questionnaire measuring adaptive and maladaptive coping strategies, alongside disaster exposures and pregnancy complications. They reported that higher perceived stress (aOR = 1.50; 95% CI = 1.34–1.99, *P* < 0.01) was significantly associated with induction of labour, and that perceived stress predicted pregnancy-induced hypertension (aOR = 1.16; 95% CI = 1.05–1.30, *P* < 0.01). Although this cross-sectional/ prospective study (with comparison being lower exposure/no cases of high perceived stress) emphasised pregnancy complications rather than directly measuring PTSD or depression, it provides support that disaster-mediated psychosocial stress (and coping style) in pregnancy is associated with adverse maternal health outcomes, which in turn may be mediated through psychological distress. An investigation on the 2008 Iowa Floods (USA) in pregnant women, using a prospective perinatal cohort, reinforces the inference that subjective reaction to disaster in pregnancy is a key mechanistic link to perinatal mood outcomes. Using a prospective perinatal cohort, the authors found that peritraumatic distress mediated the effect of flood severity on perinatal depression. Specifically, greater flood exposure severity was associated with higher peritraumatic distress, which in turn predicted higher maternal depressive symptoms. The mediation effect was statistically significant (β ≈ 0.16; 95% CI = 0.04–0.30) [26]. In a population-based study of Hurricane Michael (USA), Harville and colleagues [27] investigated adverse social and mental-health risk factors among pregnant women pre/post storm. The design compared exposures among participants before and after the disaster, and found significant increases in anxiety, depression screening scores, and social risk factors in the post-event period (*e.g*. elevated depression screening: aOR ≈ 1.4; 95% CI = 1.1–1.8, *P* < 0.01). This study further contributes to the evidence that disaster exposure during or around pregnancy is associated with elevated maternal mental-health burden. Simeone and colleagues [10] analysed data from the 2018 Puerto Rico PRAMS hurricane to examine how experiences during and after Hurricanes Irma and Maria related to postpartum depressive symptoms among women with a recent live birth. Overall, 13.7% of women reported postpartum depressive symptoms, with a higher prevalence among those who had been pregnant at landfall (15.6%) than those who conceived during recovery (8.3%).

In the Australian context, the Queensland Flood Study [25] of 183 pregnant women exposed to the 2011 Queensland floods (Australia) illustrates the importance of subjective components (*i.e.* peritraumatic distress, cognitive appraisal) in the disaster-mental-health pathway for pregnant women, beyond mere objective exposure. The researchers employed a longitudinal design, measuring disaster exposure within a year of the flood and maternal depression/anxiety at several follow-up time points (16 months, 30 months, four years, and six years postpartum). They found that objective hardship from the flood did not significantly predict maternal depression or anxiety trajectories, but peritraumatic distress did predict worse depression and anxiety only when cognitive appraisal was negative. Specifically, in linear mixed model analyses, the interaction of peritraumatic distress and negative cognitive appraisal was significant for anxiety (β = 0.52; standard error = 0.15, *P* < 0.001) and depression (β = 0.55; standard error = 0.17, *P* < 0.01), but when cognitive appraisal was neutral/positive, the effect was essentially ‘buffered’ [25]. Another study in Australia on cyclones, Parayiwa and colleagues [15] surveyed 90 pregnant women in cyclone-affected Queensland and examined factors influencing perceived stress measured by the Perceived Stress Scale-10. Using hierarchical linear regression, they found that levels of perceived stress were significantly higher for women born in New Zealand who experienced cyclone stressors (β = 0.48; *P* = 0.014), UK/Western Europe born women experiencing non-cyclone stressors (β = 0.44; *P* = 0.014), and those experiencing pregnancy complications (β = 0.43; *P* = 0.011). This suggests that disaster-related stress among pregnant women is modulated by demographic/vulnerability factors such as migrant background. Although the study did not directly measure clinical depression or PTSD, it does provide evidence of elevated stress as an outcome of disaster exposure in pregnancy.

#### Earthquake and maternal mental health

Results from eight earthquake-focused studies from China (n = 3), Japan (n = 3), Nepal (n = 1), and Türkiye (n = 1) shows that exposure to seismic disasters during pregnancy was consistently associated with elevated symptoms of depression, PTSD, and psychological distress, although effect magnitudes and persistence varied by timing, context, and comparator group (**Table 1**). In China, studies following the 2008 Sichuan and 2013 Lushan earthquakes in Japan showed substantial psychological burden during pregnancy. Qu and colleagues [28] reported major depression in 40.8% (95% CI = 35.5–46.4) and PTSD in 12.2% (95% CI = 9.0–16.4), with bereavement and witnessing entrapment nearly doubling PTSD risk (odds ratio (OR) = 1.80; 95% CI = 1.43–2.26). Pregnancy-related stress increased both depression and PTSD (OR = 1.19; 95% CI = 1.12–1.27), while family support was protective. Follow-up work a year later [29] showed similarly high depression rates in both earthquake-affected and unaffected areas, indicating that long-term psychological risk was driven more by ongoing psychosocial stress and reduced partner support than by residual earthquake exposure [29]. Ren and colleagues [30] surveyed 128 pregnant women at the Lushan epicentre and found a depression incidence of 35.2%. This study further demonstrated that, immediately post-quake, injured relatives, low support, and negative coping heightened depressive symptoms, whereas positive coping buffered risk (all *P* < 0.05).

Japanese data reinforce and extend these patterns. In the Noto Peninsula earthquake, Hibino and colleagues [31] showed that 13.1% of pregnant and postpartum women exceeded EPDS≥9, higher than non-disaster Japanese estimates, and that pre-existing earthquake-related anxiety significantly predicted antenatal/postnatal depressive symptoms (β = 0.27; *P* = 0.01). Stress resilience moderated this effect (β = −0.21; *P* = 0.02), and higher antenatal EPDS scores predicted pregnancy/birth complications (OR = 1.21; 95% CI = 1.04–1.41), demonstrating clinically meaningful vulnerability. Following the 2011 Great East Japan Earthquake, large Japan Environment and Children's Study cohorts further showed sustained distress in Miyagi for up to three years, with adjusted ORs between 1.38 and 1.92 compared with less affected regions [16]. Watanabe and colleagues [33] similarly found higher distress in Miyagi (aOR = 1.49; 95% CI = 1.06–2.09) and coastal tsunami (aOR = 1.68; 95% CI = 1.00–2.82) areas, although these regional effects were attenuated after adjusting for disaster-related life events which strongly predicted distress (OR = 3.82; 95% CI = 2.86–5.12). Together, Japanese evidence highlights both acute psychological disruption and the mediating influence of secondary stressors such as bereavement, relocation, and prolonged uncertainty.

In Nepal, six months after the 2015 earthquakes, Khatri and colleagues [32] found common mental disorder symptoms in 21.9% (95% CI = 18.4–25.8) of pregnant women, with earthquake damage and intimate partner violence increasing symptoms, and supportive partnerships and income-generating work offering protection (all *P* < 0.05). Early post-disaster data from Türkiye’s 2023 Kahramanmaraş earthquakes show strong correlations between depression, anxiety, stress and PTSD in pregnant and postpartum women living in tent cities (all *P* < 0.001), indicating severe acute distress even without reported effect sizes [6].

#### Wildfire and maternal mental health

Exposure to wildfire was associated with substantial psychological burden among pregnant women (**Table 1**). In the Fort McMurray Wood Buffalo, Alberta (Canada) wildfire in 2016, a comparative longitudinal observational study on pregnant or peri-conception women [34] was conducted, in which 23.5% met criteria for possible PTSD and 26% for probable PTSD based on Impact of Event Scale-Revised thresholds. Peritraumatic distress and dissociation were strongly associated with PTSD-like symptoms (r = 0.56; *P* < 0.001), and in multivariable models, peritraumatic distress remained a significant predictor of symptom severity. Social-support satisfaction showed a protective effect (β = 7.08; 95% CI = 2.29–11.87, *P* = 0.004) and significantly moderated the association between distress and PTSD-like symptoms (β = 0.217; 95% CI = 0.049–0.386, *P* = 0.012). Although this study lacked an unexposed control group, the internal contrasts provide robust quantitative evidence that wildfire-related psychological stress in pregnancy is clinically meaningful and statistically significant.

### Man-made disaster and maternal mental health

#### Conflict/war/terrorism and maternal mental health

Results from five studies showed a high burden of maternal mental ill-health in pregnant women exposed to organised violence, with clinically important effect sizes that remain significant at conventional 5% thresholds (**Table 2**). Among internally displaced Yazidi women in northern Iraq, direct survivors of genocidal attacks, Seidi and colleagues [35] conducted a descriptive cross-sectional study of 122 pregnant and ≤1-year postpartum women living in camps, using the EPDS to screen for perinatal depressive symptoms. Overall, 78% scored above EPDS>10 and 53% above EPDS>12, indicating moderate to severe symptomatology. Logistic regression (α = 0.05) showed that pregnancy-related health problems tripled the odds of elevated depression risk (OR = 3.22; 95% CI = 1.08–9.61), while older age (OR = 0.81; 95% CI = 0.73–0.91) and higher education (OR = 0.11; 95% CI = 0.03–0.35) were protective, and being unmarried carried a very large but imprecise risk estimate (OR = 16.00; 95% CI = 1.14–224.28).

In post-conflict Timor-Leste, a structural equation modelling analysis was conducted on cross-sectional data from 427 women 3–6 months pregnant or postpartum, defining depression as EPDS≥13 and PTSD as Harvard Trauma Questionnaire ≥2.0. Of all participants, 22% met the depression cut-off and 9% met the PTSD threshold. All model paths were significant (*P* < 0.05), with a strong path from PTSD to depressive symptoms (β = 0.40) and paths to continuing adversity depression (β = 0.22) and intimate partner violence depression (β = 0.17) [37].

Community-based data from Pakistan similarly quantify conflict-related distress in pregnancy. In Swat, a cross-sectional survey of 349 pregnant women one-year post-conflict used the Self-Reporting Questionnaire-20 (cut-off ≥9), identifying psychological distress in 38.1% (95% CI = 33.1–43.3) of participants. Distress rose sharply with cumulative conflict-related posttraumatic events, women with ≥3 posttraumatic events had over twice the odds of distress compared to those with ≤2 (OR = 2.62; 95% CI = 1.22–5.61), and ≥3 major life events in the post-conflict year increased odds more than 3-fold (OR = 3.25; 95% CI = 1.82–5.82). Perceived family support exerted a small but significant protective effect (OR = 0.91, 95% CI = 0.88–0.95) [13]. A related case-control study from the same conflict-affected region (Malakand) enrolled 225 LBW infants and 225 normal-weight controls within 24 hours of delivery, assessing maternal PTSD with the Mini Neuropsychiatric Interview 5.0. In this study, PTSD was significantly more common among mothers of LBW infants (31.6%) than controls (5.8%) (OR = 7.52; 95% CI = 4.02–14.07), and remained independently associated with LBW after adjustment for education, parity, inter-pregnancy interval, and prior preterm birth (α = 0.05) [36].

A retrospective time-series analysis of maternity hospital records in Kuwait (1981–1995) compared obstetric patterns before and after the Gulf War, inferring the effects of war-related environmental, social and psychological stress. Although maternal mental health was not directly measured, the post-invasion period showed significant increases in pregnancy-induced hypertension, pre-eclampsia, hysterectomy for postpartum haemorrhage, spontaneous abortion, and LBW births, alongside shifts in maternal age and parity. The authors explicitly attributed these adverse trends partly to war-related anxiety and stress [38].

#### Plane crash and maternal mental health

Evidence from a natural experiment within the Dutch Holistic Approach to Pregnancy and the First Postpartum Year pregnancy cohort indicates that the MH17 plane crash was followed by a short-term rise in depressive symptoms among pregnant women (**Table 2**). Those pregnant in the month after the crash had higher depressive scores at 32 weeks (mean = 5.21 vs 4.11; *t* = 2.12; *P* = 0.03; d ≈ 0.3), and the association remained significant after adjustment (*P* = 0.02). Although more women exceeded the clinical cut-off (15.9% vs 8.8%), this was not statistically significant (*P* = 0.11). The findings suggest that highly publicised plane-crash events can transiently elevate depressive symptoms in late pregnancy, with effects detectable at conventional significance thresholds [39].

#### Nuclear disaster and maternal mental health

Following the Fukushima Daiichi nuclear accident, large population-based surveys showed a marked rise in maternal depressive symptoms. In two cohorts of mothers giving birth in 2012 (n = 6686) and 2013 (n = 6423), 25% screened positive for depression. Evacuation independently predicted elevated depressive symptoms in 2012 (aOR = 1.60; 95% CI = 1.30–1.99) and 2013 (aOR = 1.44; 95% CI = 1.14–1.82), and radiation-related concern showed even stronger associations in both 2012 (aOR = 3.41; 95% CI = 2.59–4.50) and 2013 (aOR = 2.32; 95% CI = 1.44–3.76), with all associations statistically significant (*P* < 0.01). Disaster-related factors did not predict parenting confidence after adjustment [40] (**Table 2**).

#### Hybrid disaster and maternal mental health

The Peruvian cohort by Levey and colleagues [12] shows a high burden of antepartum PTSD (**Table 3**). Among 3372 pregnant women, 41.8% of those exposed to cumulative trauma met PTSD criteria (PCL-C≥26). A clear dose-response was observed, with each additional traumatic event increasing PTSD odds by 46% (aOR = 1.46; 95% CI = 1.39–1.54); women with 4–6 trauma types (aOR = 2.58; 95% CI = 2.16 –3.07), and ≥7 types (aOR = 9.33; 95% CI = 5.82–14.97). Interpersonal trauma showed the strongest associations (*e.g.* adult sexual assault aOR = 4.06; childhood abuse aOR = 3.43), while structural trauma, including disasters, had smaller but significant effects (aOR = 1.39; 95% CI = 1.15–1.67).

## DISCUSSION

### Key findings and interpretation

Across 24 disasters spanning natural, technological, radiological, and conflict-related events, we show that maternal mental health responses are neither uniform nor solely driven by exposure intensity. Instead, outcomes appear shaped by three interlinked mechanisms: whether the hazard is acute or protracted; the sociopolitical and health-system context of pregnancy; and women’s ability to maintain social, material, and relational support.

First, disaster type matters. Earthquakes produced high immediate psychological burden but shorter-lived effects, likely reflecting their acute, time-bounded nature, as once aftershocks cease and environments stabilise, perceived threat declines. In contrast, floods and hurricanes showed more persistent depression and stress, consistent with their slow-onset, cascading impacts, including displacement, prolonged rebuilding, financial strain, and cumulative stressors. Evidence from Queensland and Iowa [25,26] illustrates how early peritraumatic distress interacts with ongoing disruption to sustain symptoms well beyond the initial event.

Second, contextual conditions strongly influence effect magnitude. Conflict-affected settings (*e.g.* Iraq, Pakistan, and Timor-Leste) showed the highest levels of depression and PTSD (aORs ranging from 3 to 16), driven by limited antenatal mental-health services, poverty, food insecurity, chronic violence, displacement, and restricted mobility. In high-income contexts (*e.g.* MH17, Fort McMurray, and Fukushima), symptom elevations were present but typically smaller, likely reflecting stronger health systems, social protection, and accessible psychosocial care.

Third, social support is protective but not uniformly so – its buffering effect depends on timing, distress severity, and access to material resources. In wildfire settings, social support reduced PTSD only at low-to-moderate distress levels, suggesting that once distress exceeds a threshold, relational buffering is overwhelmed. In conflict environments, where partner support may be compromised by displacement, insecurity, or intimate partner violence, community-based support may be more relevant than household support. Thus, social support is neither conceptually uniform nor universally effective; its value varies by structure, accessibility, and cultural norms.

Radiological and hybrid disasters highlight persistent uncertainty. In Fukushima [40], evacuation and radiation-related worry strongly predicted depressive symptoms even after physical conditions stabilised, reflecting chronic ambiguity about safety and foetal risk. Similarly, in Peru’s hybrid disaster environment [12], cumulative trauma, not the natural hazard alone, produced extremely high PTSD burden. These patterns suggest that uncertainty, threat duration, and trauma complexity may be more predictive of mental-health trajectories than physical hazard type.

Collectively, these findings challenge the assumption that disasters uniformly worsen maternal mental health. Impact depends on how the event unfolds, institutional response, and the stability of women’s social and economic supports. Interventions must therefore be tailored – trauma-focused care after acute events, sustained psychosocial and resource-oriented support after hydrometeorological disasters, protection from gender-based violence and chronic adversity in conflict settings, and long-term risk-communication strategies after nuclear or hybrid disasters. Ultimately, maternal mental health outcomes reflect the interaction between pregnancy-related biological vulnerability and the broader social ecology of disaster exposure, underscoring the need for integrated, trauma-informed, context-specific approaches across all phases of disaster preparedness and response.

### Implications for practice and policy

We highlight the need for maternal mental health to be embedded in clinical care and disaster-response planning. Pregnant women consistently showed elevated risks of depression, anxiety, PTSD, and psychological distress across disaster types, underscoring the importance of routine screening with validated, context-appropriate tools and clear referral pathways.

For policymakers, integrating maternal mental health into emergency preparedness is essential. Priorities include continuity of antenatal and psychosocial care, safe shelter and basic resources during displacement, and access to evidence-based mental health services. Given variability in study quality and the absence of evidence-certainty grading, policy decisions should remain cautious while emphasising scalable, low-cost strategies – such as community-based psychosocial support, task-shared services, and family or partner engagement – as higher-quality evidence continues to emerge.

### Limitations

This review has several limitations. Considerable heterogeneity in study designs, exposure definitions, mental health measures, and assessment timeframes prevented a meta-analysis and limited comparability across studies. Most studies used cross-sectional designs and several lacked full statistical reporting, such as adjusted estimates or CIs, restricting causal inference and weakening interpretation. Mental health instruments were applied across varied cultural and linguistic contexts, which may influence symptom reporting. Key confounders, including pre-existing mental health conditions, socioeconomic status, displacement, and access to care, were not consistently measured or adjusted for, and some studies did not disaggregate data specifically for pregnant women. Since we focused on English-language, peer-reviewed publications, there may be a language and publication bias, and exclusion of grey literature or unpublished studies may have resulted in omissions. Finally, we focused solely on quantitative evidence and did not formally grade overall evidence certainty, so findings should be interpreted with appropriate caution.

## CONCLUSIONS

Our results show that disasters, whether natural, technological, radiological, or conflict-related, significantly heighten the risk of depression, anxiety, PTSD, and psychological distress among pregnant women. The severity and duration of these effects are shaped not just by the hazard, but by how long the threat persists, the cumulative trauma women face, and the stability of their social, economic, and healthcare environments. Pregnancy emerges as a uniquely vulnerable period in which biological sensitivity and concern for foetal safety intensify psychological impact.

Our findings position maternal mental health as essential to disaster preparedness and response. Routine screening, trauma-informed antenatal care, continuity of maternity services, and targeted support for displaced or highly exposed women must become core components of disaster-health planning. Clear communication and protection from gender-based violence are especially critical in prolonged or uncertainty-driven events, such as radiological or hybrid disasters. Future research should strengthen longitudinal and comparative designs, harmonise outcome measures, and further examine mediating factors, such as peritraumatic distress, social support, and institutional response. Safeguarding maternal mental health in disaster settings is both a public-health priority and an equity obligation. Supporting pregnant women before, during, and after disasters is vital not only for their well-being, but for the resilience and health of future generations.

There is a need for more robust longitudinal studies using standardised mental health measures and complete statistical reporting, as much of the existing evidence is cross-sectional and insufficiently adjusted for confounding. Rigorous quasi-experimental and natural-experiment designs should also be prioritised to strengthen causal inference. Disaster impacts in low-income, conflict-affected, and recurrent-disaster settings remain underrepresented, and targeted research in these contexts is essential for global relevance. Future studies should also clarify pathways linking disaster exposure to maternal and child outcomes, including chronic stress, displacement, cumulative trauma, and variations in social support. Preregistration, transparent confounder control, and full reporting of effect sizes and CIs should also be implemented, enabling stronger evidence grading and more effective translation into policy and practice.

## Additional material


Online Supplementary Document
