# Supplementary material for: The impact of disasters on maternal mental health: a systematic review
Source: J Glob Health. 2026 Apr 17;16:04126. doi: 10.7189/jogh.16.04126 (PMC13086487; doi:10.7189/jogh.16.04126)
Supplement: Online Supplementary Document [file jogh-16-04126-s001.pdf]

Supplement to: Seema M, Afoakwah C, Tuzel E, Byrnes J. The impact of disasters on maternal mental health: a systematic review. J Glob Health. 2026;16:04126.

**Table S1. Databases and Sources Used for the Literature Search**			
**S.No**	**Database / Source**	**Platform / Provider**	**Notes / Sub-databases (if applicable)**
1	MEDLINE	Ovid	Covers PubMed-indexed biomedical literature
2	PsycINFO	Ovid	Psychology and behavioural sciences
3	CINAHL	EBSCOhost	Nursing & allied health literature
4	Embase	Elsevier	Biomedical & pharmacological literature
5	Scopus	Elsevier	Multidisciplinary; includes ScienceDirect content
6	Web of Science Core Collection	Clarivate	Multidisciplinary academic database
7	ProQuest Central	ProQuest	Multidisciplinary mega-database; includes sub-collections below
7a	– ABI/INFORM Collection	ProQuest	Business & management
7b	– Consumer Health Database	ProQuest	Consumer-oriented health sciences
7c	– Health & Medical Collection	ProQuest	Clinical and public health research
7d	– Healthcare Administration Database	ProQuest	Health systems, administration, policy
7e	– Nursing & Allied Health Database	ProQuest	Professional nursing & allied health content
7f	– Psychology Database	ProQuest	Psychological research
7g	– Public Health Database	ProQuest	Epidemiology & public health literature
7h	– Social Science Database	ProQuest	Social policy, sociology, behavioural sciences

7i	– Sociology Database	ProQuest	Focused sociology content
8	EconLit	EBSCOhost	Economics & health economics
9	JSTOR	JSTOR	Archival academic literature across disciplines
10	PTSDpubs	ProQuest / EBSCOhost	PTSD and trauma-related literature
11	Sociological Abstracts	ProQuest	Sociology and social theory
12	International Bibliography of the Social Sciences (IBSS)	ProQuest	Social sciences & interdisciplinary research
13	Google Scholar	Web search engine	Supplementary grey-literature and citation search

**Table S2. Search Strategy for Medline Total hits = 4718** (adapted in all the other databases)	
S/n	Searches
1.	Maternal*ti,ab,kw.
2.	Mother*ti,ab,kw
3.	exp *Maternal Health/
4.	exp *Mothers/
5.	(Maternal* or Mother* or Prenatal* or Pre-natal* or Perinatal* or Antenatal* or Ante-natal or Human females* or Pregnant* or Post-partum).ti,ab,kw.
6.	1 or 2 or 3 or 4 or 5
7.	exp *Mental Health/
8.	exp *Depression/ or exp *Psychiatry/ or exp *Mental Disorders/ or exp *Humans/ or exp *Female/ or exp *Stress/or exp *Anxiety/ or exp *PTSD/ or * 'post traumatic stress disorder'
9.	((mental* or psychological*) adj5 (health* or wellbeing* or 'well being*' or 'well-being*' or wellness* or fitness* or illness or stress or 'mood disorder*')).ti,ab,kw.
10.	7 or 8 or 9
11.	exp *Natural Disasters/ or exp *Disasters/
12.	((natural or environmental or ecological or 'man made' or manmade or 'man-made' or artificial or unnatural or industrial or hybrid or nuclear) adj5 (disaster* or calamity* or catastrophe* or emergency or crisis)).ti,ab,kw.
13.	('bush fire*' or bushfire* or wildfire* or 'wild fire*' or forestfire* or 'forest fire*' or drought* or flood* or 'extreme weather' or earthquake* or 'earth quake' or cyclone* or hurricane* or femine* or tsunami* or heatwave* or 'heat wave*' or coldwave* or 'cold wave* or 'ice storm*' or 'icestorm*' or war* or conflict* or terrorist* or 'violent extremis*' or 'extremis* violence' or 'civil disorder*' or 'civil unrest*' or displacement*or 'forced migration*).ti,ab,kw.
14.	11 or 12 or 13
15.	6 and 10 and 14

**Table S3. Article Screening and Eligibility Form**			
**Stage**	**Screening Item**	**Response (Yes/No)**	**Notes (if any)**
**Stage 1: Title & Abstract Screening**	Research on maternal mental health	Yes / No	
	Original research article	Yes / No	
	Disaster-related	Yes / No	
	Human pregnant women / mothers	Yes / No	
	All Stage 1 criteria met	Yes / No	
**Stage 2: Full-Text Eligibility**	Clear disaster-related evaluation	Yes / No	
	Maternal mental health outcomes assessed	Yes / No	
	Eligible for inclusion in final extraction	Yes / No	
	Reasons for exclusion / Additional details	—	(e.g., incomplete data, mental health not measured, wrong population)

Table S4. Items on data collection form for included studies		
**Category**	**Data Item**	**Information to Extract**
**Publication Details**	Study ID	Unique ID assigned for review management
	Author(s) & Year	Full citation (e.g., Hibino et al., 2009)
	Country / Region	Study location
	Journal / Source	Publication outlet
	Funding Source	Yes/No and details if reported
**Study Characteristics**	Study Design	Cohort, cross-sectional, case-control, natural experiment, etc.
	Study Period	Data collection timeframe
	Sample Size	Analytic sample (final N)
	Participant Characteristics	Age, parity, SES indicators, inclusion/exclusion criteria
	Recruitment Method	Sampling frame, approach used
**Disaster Exposure**	Type of Disaster	CRED classification: earthquake, flood, hurricane, conflict, wildfire, etc.
	Exposure Timing	Trimester, gestational week, or pregnancy stage at exposure
	Exposure Definition	Objective (e.g., proximity, intensity, damage) or subjective (perceived threat, distress)
	Measurement Method	Scale/index, GIS mapping, external databases, government categories
	Comparison Group	Unexposed or lower-exposure group characteristics (if applicable)
**Maternal Mental Health Outcomes**	Outcome Category	Depression, anxiety, stress, PTSD, psychological distress
	Measurement Tool	EPDS, DASS, K6, PCL, CES-D, or validated scale
	Timing of Assessment	During pregnancy or postpartum
	Scoring Approach	Continuous score, cutoff-based, severity categories
**Effect Estimates**	Summary Statistic	Prevalence, mean, SD, baseline result
	Effect Measure	Adjusted OR, RR, HR, β-coefficients, mean difference, SMD
	Covariates Adjusted	Confounders included in analysis
	Confidence Intervals	95% CI
	P-values	If reported

	Direction & Magnitude	Short description of effect pattern
**Risk of Bias & Quality**	JBI/Quality Score	Risk of bias outcome
	Methodological Limitations	Missing data, small sample, inadequate adjustment, etc.
**Additional Notes**	Contextual Factors	Displacement, loss, injury, social support, healthcare access
	Pregnancy Outcomes (if relevant)	Preterm birth, LBW, gestational complications
	Reviewer Notes	Clarifications or extraction issues

Notes:
CRED = Centre for Research on the Epidemiology of Disasters classification.
SES = Socioeconomic status.
EPDS = Edinburgh Postnatal Depression Scale.
DASS = Depression Anxiety Stress Scales.
K6 = Kessler Psychological Distress Scale.
PCL = Post-Traumatic Stress Disorder Checklist.
CES-D = Center for Epidemiologic Studies Depression Scale.
PTSD = Post-traumatic stress disorder.
OR/AOR = (Adjusted) Odds Ratio.
RR/ARR = (Adjusted) Relative Risk.
HR = Hazard Ratio.
SMD = Standardised Mean Difference.
CI = Confidence Interval.
β-coefficient = Regression coefficient representing magnitude of association.
Additional clarifications:

- "Exposure Timing" refers to the gestational stage during which the disaster occurred.
- "Validated tools" refer to standardised, psychometrically tested mental health scales.
- "Effect measures" prioritise adjusted estimates to minimise confounding.
- JBI = Joanna Briggs Institute Critical Appraisal tool.

Table S5. Joanna Briggs Institute (JBI) critical appraisal results

JBI Critical Appraisal Checklist for systematic reviews and research syntheses

Reviewers: Minnat Seema, Clifford Afoakwah, Elcin Tuzel, Joshua Byrnes

Date: October 1, 2023, to May 31, 2025

Author: Minnat Seema, Clifford Afoakwah, Elcin Tuzel, Joshua Byrnes

Year: October 1, 2023, to May 31, 2025. Record Number: Study #1

	Yes	No	Unclear	Not applicable
1. Is the review question clearly and explicitly stated?	▯	☐	☐	☐
2. Were the inclusion criteria appropriate for the review question?	▯	☐	☐	☐
3. Was the search strategy appropriate?	▯	☐	☐	☐
4. Were the sources and resources used to search for studies adequate?	▯	☐	☐	☐
5. Were the criteria for appraising studies appropriate?	▯	☐	☐	☐
6. Was critical appraisal conducted by two or more reviewers independently?	▯	☐	☐	☐
7. Were there methods to minimize errors in data extraction?	▯	☐	☐	☐
8. Were the methods used to combine studies appropriate?	▯	☐	☐	☐
9. Was the likelihood of publication bias assessed?	▯	☐	☐	☐

10. Were recommendations for policy and/or practice supported by the reported data? ▯ □ □ □

11. Were the specific directives for new research appropriate? ▯ □ □ □

Overall appraisal: Include ▯ Exclude ▯ Seek further info □

Comments: Including -Study meets inclusion criteria, pregnant women exposed to hurricane/flood; valid mental health outcome measure; acceptable methodological quality. Excluded: mental health outcomes not reported separately for pregnant women

© JBI, 2020. All rights reserved. JBI grants use Critical Appraisal Checklist for Systematic Reviews and Research Syntheses - **1**of these tools for research purposes only. All other enquiries should be sent to jbisynthesis@adelaide.edu.au

Table S6. PRISMA 2020 checklist

Section and Topic	Ite m #	Checklist item	Location where item is reported
**TITLE**			
Title	1	Identify the report as a systematic review.	Title page
**ABSTRACT**			
Abstract	2	See the PRISMA 2020 for Abstracts checklist.	Abstract
**INTRODUCTION**			
Rationale	3	Describe the rationale for the review in the context of existing knowledge.	Background
Objectives	4	Provide an explicit statement of the objective(s) or question(s) the review addresses.	why this review is important, objectives
**METHODS**			
Eligibility criteria	5	Specify the inclusion and exclusion criteria for the review and how studies were grouped for the syntheses.	Methods
Information sources	6	Specify all databases, registers, websites, organisations, reference lists and other sources searched or consulted to identify studies. Specify the date when each source was last searched or consulted.	Methods, eligibility criteria
Search strategy	7	Present the full search strategies for all databases, registers and websites, including any filters and limits used.	Methods, search strategy
Selection process	8	Specify the methods used to decide whether a study met the inclusion criteria of the review, including how many reviewers screened each record and each report retrieved, whether they worked independently, and if applicable, details of automation tools used in the process.	Methods, selection of studies
Data collection process	9	Specify the methods used to collect data from reports, including how many reviewers collected data from each report, whether they worked independently, any processes for obtaining or confirming data from study investigators, and if applicable, details of automation tools used in the process.	Methods, data extraction
Data items	10a	List and define all outcomes for which data were sought. Specify whether all results that were compatible with each outcome domain in each study were sought (e.g. for all measures, time points, analyses), and if not, the methods used to decide which results to collect.	Methods, outcomes
	10b	List and define all other variables for which data were sought (e.g. participant and intervention characteristics, funding sources). Describe any assumptions made about any missing or unclear information.	Methods, data extraction
Study risk of bias assessment	11	Specify the methods used to assess risk of bias in the included studies, including details of the tool(s) used, how many reviewers assessed each study and whether they worked independently, and if applicable, details of automation tools used in the process.	Methods, data extraction
Effect measures	12	Specify for each outcome the effect measure(s) (e.g. risk ratio, mean difference) used in the synthesis or presentation of results.	Method, data synthesis, the measure of effect
Synthesis methods	13a	Describe the processes used to decide which studies were eligible for each synthesis (e.g. tabulating the study intervention characteristics and comparing against the planned groups for each synthesis (item #5)).	Method
	13b	Describe any methods required to prepare the data for presentation or synthesis, such as handling of missing summary statistics, or data conversions.	Data synthesis, the measure of effect
	13c	Describe any methods used to tabulate or visually display results of individual studies and syntheses.	Data synthesis, the measure of effect
	13d	Describe any methods used to synthesize results and provide a rationale for the choice(s). If meta-analysis was performed, describe the model(s), method(s) to identify the presence and extent of statistical heterogeneity, and software package(s) used.	Data synthesis
	13e	Describe any methods used to explore possible causes of heterogeneity among study results (e.g. subgroup analysis, meta-regression).	Data synthesis, Subgroup
	13f	Describe any sensitivity analyses conducted to assess robustness of the synthesized results.	Data synthesis, sensitivity analysis
Reporting bias assessment	14	Describe any methods used to assess risk of bias due to missing results in a synthesis (arising from reporting biases).	Data extraction
Certainty	15	Describe any methods used to assess certainty (or confidence) in the body of evidence for an outcome.	Data extraction

Section and Topic	Item #	Checklist item	Location where item is reported
assessment			
**RESULTS**			
Study selection	16a	Describe the results of the search and selection process, from the number of records identified in the search to the number of studies included in the review, ideally using a flow diagram.	Results
	16b	Cite studies that might appear to meet the inclusion criteria, but which were excluded, and explain why they were excluded.	Results
Study characteristics	17	Cite each included study and present its characteristics.	Results
Risk of bias in studies	18	Present assessments of risk of bias for each included study.	Results, Study characteristics and risk of bias
Results of individual studies	19	For all outcomes, present, for each study: (a) summary statistics for each group (where appropriate) and (b) an effect estimate and its precision (e.g. confidence/credible interval), ideally using structured tables or plots.	Results
Results of syntheses	20a	For each synthesis, briefly summarise the characteristics and risk of bias among contributing studies.	Results, Study characteristics and risk of bias
	20b	Present results of all statistical syntheses conducted. If meta-analysis was done, present for each the summary estimate and its precision (e.g. confidence/credible interval) and measures of statistical heterogeneity. If comparing groups, describe the direction of the effect.	N/A
	20c	Present results of all investigations of possible causes of heterogeneity among study results.	Results
	20d	Present results of all sensitivity analyses conducted to assess the robustness of the synthesized results.	N/A
Reporting biases	21	Present assessments of risk of bias due to missing results (arising from reporting biases) for each synthesis assessed.	Results, Study characteristics and risk of bias
Certainty of evidence	22	Present assessments of certainty (or confidence) in the body of evidence for each outcome assessed.	Table 1, Table 2 & Table 3
**DISCUSSION**			
Discussion	23a	Provide a general interpretation of the results in the context of other evidence.	Discussion
	23b	Discuss any limitations of the evidence included in the review.	Discussion
	23c	Discuss any limitations of the review processes used.	Discussion
	23d	Discuss implications of the results for practice, policy, and future research.	Discussion
**OTHER INFORMATION**			
Registration and protocol	24a	Provide registration information for the review, including register name and registration number, or state that the review was not registered.	Abstract, registration
	24b	Indicate where the review protocol can be accessed, or state that a protocol was not prepared.	Protocol wasn't published
	24c	Describe and explain any amendments to information provided at registration or in the protocol.	N/A
Support	25	Describe sources of financial or non-financial support for the review, and the role of the funders or sponsors in the review.	Grant information
Competing interests	26	Declare any competing interests of review authors.	Competing interest
Availability of data, code and other materials	27	Report which of the following are publicly available and where they can be found: template data collection forms; data extracted from included studies; data used for all analyses; analytic code; any other materials used in the review.	Search strategy

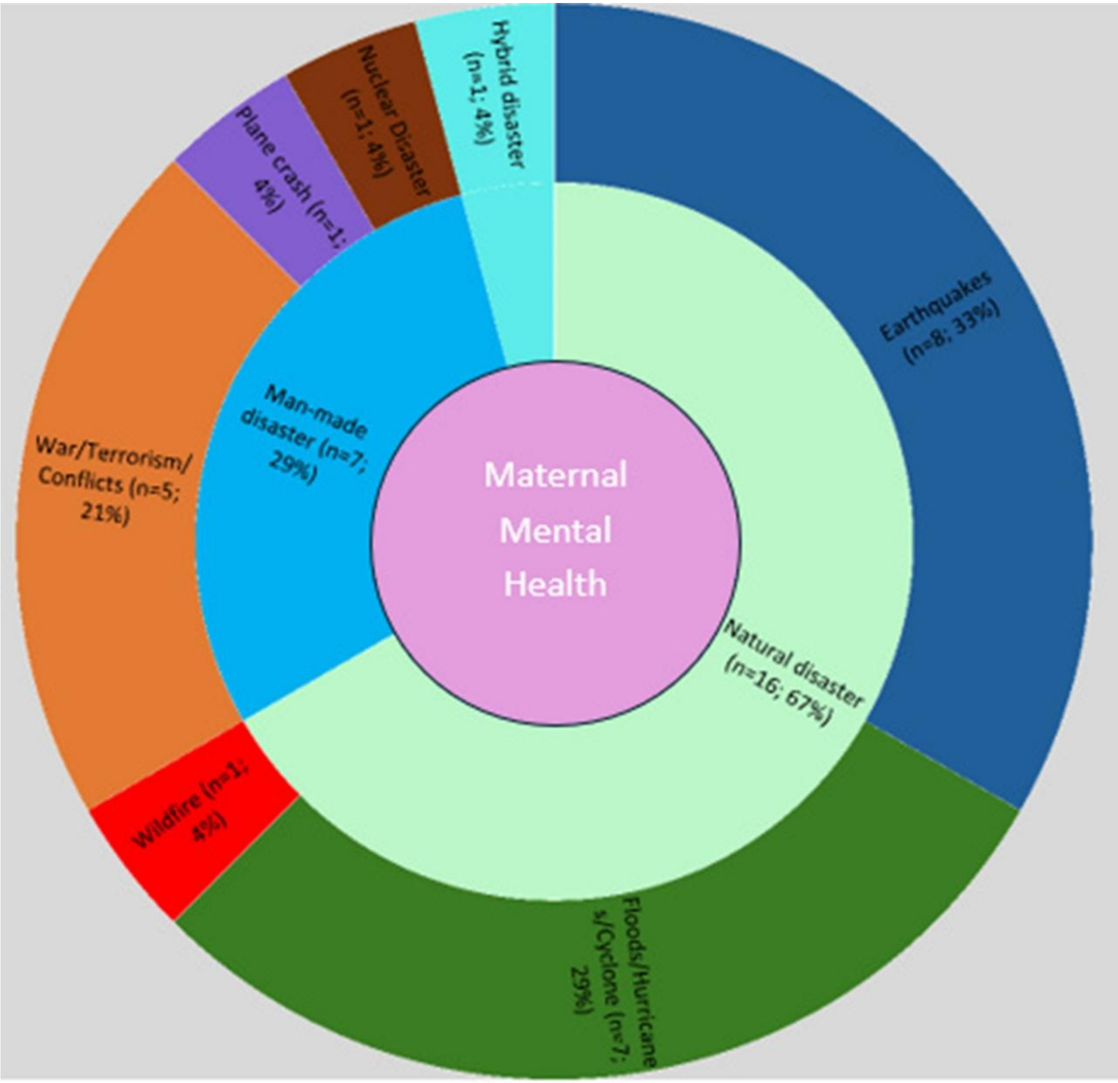

Figure S1. Distribution of the disasters in the review.
